# A Novel Huntington’s Disease Assessment Platform to Support Future Drug Discovery and Development

**DOI:** 10.3390/ijms232314763

**Published:** 2022-11-25

**Authors:** Jingyun Wu, Luisa Möhle, Thomas Brüning, Iván Eiriz, Muhammad Rafehi, Katja Stefan, Sven Marcel Stefan, Jens Pahnke

**Affiliations:** 1 Department of Pathology, Section of Neuropathology, Translational Neurodegeneration Research and Neuropathology Lab, University of Oslo and Oslo University Hospital, Sognsvannsveien 20, 0372 Oslo, Norway; www.pahnkelab.eu; 2 Institute of Clinical Pharmacology, University Medical Center Göttingen, Robert-Koch-Str. 40, 37075 Göttingen, Germany; 3 Pahnke Lab (Drug Development and Chemical Biology), Lübeck Institute of Experimental Dermatology (LIED), University of Lübeck and University Medical Center Schleswig-Holstein, Ratzeburger Allee 160, 23538 Lübeck, Germany; 4 Department of Pharmacology, Faculty of Medicine, University of Latvia, Jelgavas iela 4, 1004 Rīga, Latvia; 5 Department of Neurobiology, The Georg S. Wise Faculty of Life Sciences, Tel Aviv University, Tel Aviv 6997801, Israel

**Keywords:** Huntington’s disease, neurodegeneration, therapy, drug discovery, drug design, ABC transporters, ABCA7, ABCB1, ABCC1, polypharmacology

## Abstract

Huntington’s disease (HD) is a lethal neurodegenerative disorder without efficient therapeutic options. The inefficient translation from preclinical and clinical research into clinical use is mainly attributed to the lack of (i) understanding of disease initiation, progression, and involved molecular mechanisms; (ii) knowledge of the possible HD target space and general data awareness; (iii) detailed characterizations of available disease models; (iv) better suitable models; and (v) reliable and sensitive biomarkers. To generate robust HD-like symptoms in a mouse model, the neomycin resistance cassette was excised from zQ175 mice, generating a new line: zQ175^Δneo^. We entirely describe the dynamics of behavioral, neuropathological, and immunohistological changes from 15–57 weeks of age. Specifically, zQ175^Δneo^ mice showed early astrogliosis from 15 weeks; growth retardation, body weight loss, and anxiety-like behaviors from 29 weeks; motor deficits and reduced muscular strength from 36 weeks; and finally slight microgliosis at 57 weeks of age. Additionally, we collected the entire bioactivity network of small-molecule HD modulators in a multitarget dataset (HD_MDS). Hereby, we uncovered 358 unique compounds addressing over 80 different pharmacological targets and pathways. Our data will support future drug discovery approaches and may serve as useful assessment platform for drug discovery and development against HD.

## 1. Introduction

### 1.1. HD Pathogenesis

HD is one of the most common inherited, autosomal-dominant neurodegenerative diseases [1]. The clinical symptoms of HD include progressive involuntary movements (chorea major), increasing cognitive impairment, and variable psychiatric disturbances [2]. The extreme intensive nursing and home care requirements for HD patients exhibit a significant socioeconomic burden with unmet medical needs. The disease is caused by the expansion of polyglutamine (polyQ) encoded by a CAG repeat region in exon 1 of the huntingtin gene (*HTT*) [1,3]. Under normal physiological conditions, the number of CAG repeats lies between 16 and 35 [2]. People who have 36 and more CAG repeats usually develop HD during their natural lifespan [2]. More than 60 CAG repeats in the *HTT* gene can lead to a juvenile onset of HD [4]. Clinical studies of HD patients demonstrated a strong correlation between the CAG repeat length and disease severity, as well as an inverse correlation with respect to age of HD onset [2,5]. HD is fatal, affecting approximately 13.7 per 100,000 individuals in the European and 5.7 per 100,000 in the North American populations [6,7]. HD onset is most frequently observed between the 4th and 5th decades of life, and the lifespan of HD patients is significantly shortened with death occurring usually 10–25 years after the first onset of HD symptoms [7,8].

From a neuropathological perspective, HD is characterized by a selective loss of striatal medium spiny neurons (MSNs) and a forebrain atrophy across main brain structures [9]. The excessive CAG repeats result in a misfolded huntingtin protein (HTT) and conformational alterations, leading to the formation of cellular inclusions, further influencing major cellular processes involving various cell types in the brain [10]. This is ultimately followed by neurodegeneration primarily in the regions of the striatum and cerebral cortex [2]. HTT, a 350 kDa protein with a polyQ domain in its N-terminal region, is abundantly expressed in the cytoplasm. It is a highly conserved protein responsible for several biological functions, such as vesicular trafficking, cellular metabolism, as well as gene transcription and translation [11,12,13].

### 1.2. Current HD Therapy

To date, only two drugs have been approved specifically to treat HD, tetrabenazine and deutetrabenazine [14]. Moreover, many already approved drugs, such as anticholinergics, antidepressants, and antipsychotics are repurposed and used off-label to target specific HD symptoms [14,15,16,17,18,19]. These drugs focus on cerebral neurotransmitter systems, as for example, receptors of dopamine (DRs), serotonin (5-hydroxy tryptamine; 5HTRs), or N-methyl-D-aspartic acid (NMDARs) which are shared amongst other neurological disorders. However, no causative, disease-modifying treatment is to this date available to stop, slow down, or reverse the disease progression or even delay HD onset [6]. Several promising target structures apart from HTT have been identified to potentially be involved in the HD pathogenesis, including cysteine aspartases (caspases, CASPs) [2,14,15,17,20,21,22,23,24,25,26], heat shock proteins (HSPs) [2,22,27,28,29,30,31,32,33,34], histone deacetylases (HDACs) [2,14,15,17,20], phosphodiesterases (PDEs) [14,15,17,31,35,36], or sigma-receptors (σRs) [14,15,35,37], amongst several others. Many interesting drug candidates were discovered targeting these and other cerebral targets, however, the reason for their positive effect on in vitro or in vivo HD models remains unclear, and the mechanisms of action toward their primary targets still need to be elucidated [38,39]. We identified five aspects which may explain the insufficient translation of promising drug candidates into clinical success: (i) the cellular and molecular complexity of HD pathogenesis and the exact molecular mechanisms involved; (ii) the in large parts hidden bioactivity network of potential HD drug targets, interacting small molecules, as well as their bioactivities and modes of action; (iii) the inadequate description of existing disease models with regard to disease time course and symptom-related, molecular characterizations; (iv) the shortcoming in the development of better suitable disease models; and (v) a lack of reliable and sensitive biomarkers of HD onset and progression besides CAG-expansion diagnostics.

### 1.3. Previously Used HD Mouse Models

A reliable and in detail characterized HD animal model that recapitulates the neuropathological features of human HD and its molecular markers is imperative for developing preclinical, disease-modifying treatments. Numerous gene-modified mouse models of HD have been established since the first identification of the mutation in 1993 [40]. Transgenic and knock-in (KI) rodent models are mostly used to provide insight into the disease mechanisms, therapeutic target identification and validation, as well as therapeutic discovery and development [41]. Transgenic HD mice such as BACHD and YAC128 were generated by introducing the full-length mutant human *HTT* gene. These mice show a steadier progression of HD phenotypes [42,43]. However, these models are not optimal for investigating the metabolic changes during HD progression, such as body weight loss, which is a key clinical hallmark of HD. KI models have the expanded CAG repeats inserted into the mouse Huntington’s disease gene homolog locus (*Hdh*, *Htt*) and can be heterozygous or homozygous for the repeat modification [42]. Considering that homozygosity is a relatively rare condition in humans, heterozygous KI mouse models mimicking the human genetic mutation causing HD are currently most preferred for preclinical research [44]. However, most KI mouse models like HdhQ150 exhibit rather mild and slow behavioral and histopathological phenotypes, despite the extensive CAG expansion, compared to other transgenic mice.

### 1.4. zQ175 Mice and Novel HD Mice

The C57BL/6JQ175KI (zQ175) mice, which originally derived from a spontaneous expansion of the CAG repeat length in the murine CAG 140 KI line, is of interest as it is the first KI mouse model to exhibit relatively robust HD phenotypes in the heterozygous form [45]. HD-related features were observed in both heterozygous and homozygous zQ175 mice, such as decreased body weight, motor deficits, brain atrophy, disrupted brain metabolites [45,46], and loss of MSNs in the caudate-putamen. Specifically, widely distributed mutant HTT (muHTT) aggregates were detected in distinct brain regions of the caudate-putamen (CP; murine analog of the human striatum) and cortex during disease progression. In addition, striatal MSN marker proteins, postsynaptic markers, and complement activation markers were conspicuously altered at different time points in zQ175 mice [44,45].

**Figure 1:**
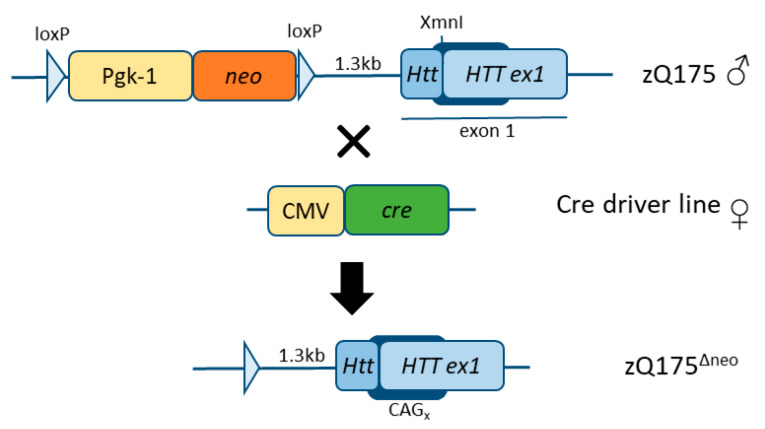
Schematic representation of the generation of the zQ175^Δneo^ mouse line (C57BL/6J background). The floxed neomycin (*neo*) resistance cassette including its promoter (Pgk-1) were initially used to select for the detection of the mutated *HTT* exon 1 insert at the *X*mnI site of the *Htt* locus. *neo* and Pgk-1 were excised between the loxP sites using a ubiquitous cre-driver mouse line where cre is expressed under the CMV promoter. The resulting locus still includes one remaining loxP site (and some bases of the initial construct which was inserted in a neighboring *H*indIII site) 1.3kb downstream of the *Htt* exon 1.

During the creation of the zQ175 mice, a strategy was used introducing a floxed neomycin (*neo*) selection cassette located approximately 1.3kb upstream of the *Htt* gene locus [45]. However, the presence of this *neo* cassette leads to alterations in the muHTT transgene expression and metabolism [44,47]. In an effort to eliminate the interference of the *neo* cassette toward the human *muHTT* insertion in exon 1 and to increase the toxicity of muHTT in this mouse model, the *neo* cassette has previously been removed from the Q175F model (FVB/N background) resulting in a more severe progression of the disease (Q175DFN) [48]. Here, we have generated and characterized another model in the C57BL/6J background, zQ175^Δneo^ (Figure 1), by crossing the former zQ175 (C57BL/6JQ175KI) line to JAX stock #006054, containing a ubiquitously expressed cre-gene, also expressed in germ lines [49].

To date, there has been a limited number of reports using zQ175-(C57BL/6J background)-related mice in behavioral and therapeutic validation studies [50,51,52,53,54,55,56,57,58]. A previous characterization study of Q175F mice (FVB/N background) revealed that these mice display an earlier and more robust phenotype with sudden death due to fatal seizures than the zQ175 line [48]. It remains to be tested if the early and enhanced HD-like neuropathological phenotypes are also apparent in zQ175^Δneo^ mice, as mice with C57BL/6J background are generally less susceptible to neurodegeneration compared to mice in the FVB/N background [59]. Our study aimed to provide a detailed neuropathological evaluation to directly compare the time-course of behavioral and neuropathological features in zQ175^Δneo^ mice, thus, establishing a novel in vivo assessment platform. To translate these findings into novel drug development, we additionally provide a complementary dataset of HD-targeting agents (HD_MDS) which serves as in silico assessment platform. Taken together, our novel HD assessment platform provides the necessary resources to promote novel therapeutic and diagnostic design, discovery, and development.

## 2. Results

### 2.1. Development, Characterization, and Establishment of a Novel HD Mouse Model

In order to increase the toxicity of muHTT and to enhance the phenotype of zQ175 mice (C57BL/6J background), we crossed the zQ175 strain with CMV-*cre* mice to remove the *neo* cassette, which otherwise potentially interferes, and subsequently, resists the inserted-transgene expression, which could further hamper a successful mouse model [60]. The resulting zQ175^Δneo^ line was characterized as follows:

#### 2.1.1. Body Weight and Food Consumption Analysis

We conducted a long-term characterization of male and female heterozygous zQ175^Δneo^ mice. From the age of 15 weeks (~4 months) to 57 weeks (~13 months), we compared different clinically relevant parameters including body weight, food consumption, motor performance, neuropsychiatric function, as well as histological and molecular features to wild-type (WT) littermates.

**Figure 2:**
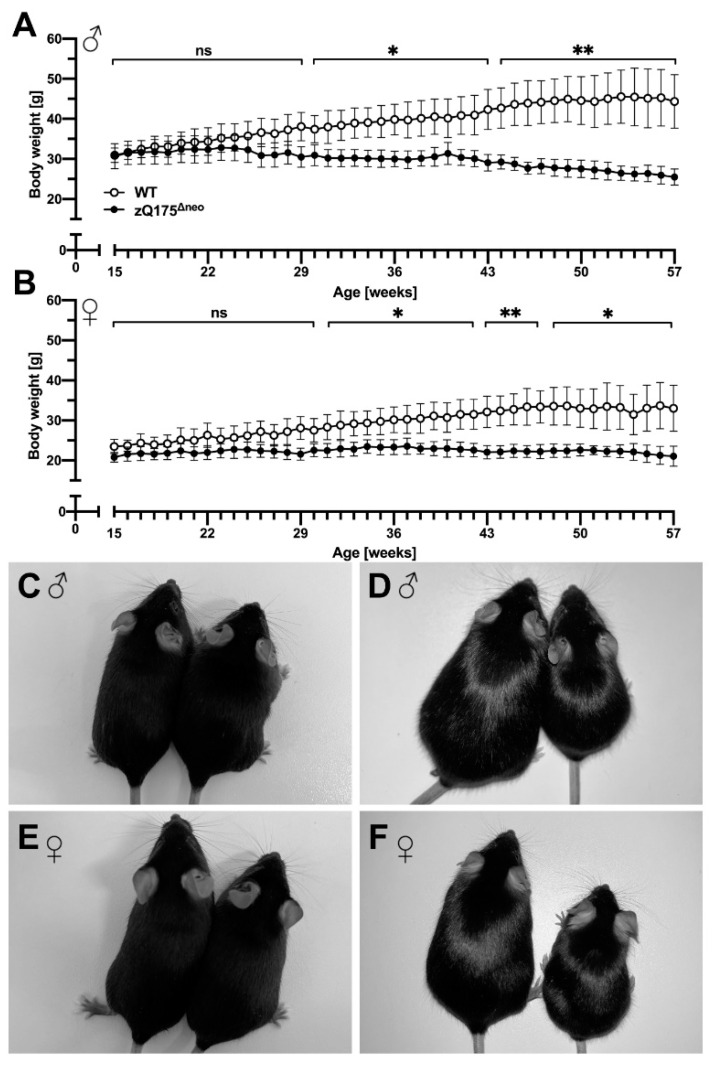
The zQ175^Δneo^ mice display a significantly reduced weight gain in the first weeks compared to WT littermates. Later on, increasing weight loss is appreciable. (**A**,**B**) The body weight of male (**A**) and female (**B**) WT (○) and zQ175^Δneo^ mice (●) was measured weekly from 15 to 57 weeks of age. Data are presented as mean ± standard deviation (SD); N = 6. Significance was calculated using multiple unpaired *t*-tests followed by Holm-Šídák correction and is indicated as ns: not significant, *: *p* ≤ 0.05, and **: *p* ≤ 0.01. (**C**–**F**) Representative images showing a direct comparison of 15-week-old (**C**,**E**) and 57-week-old (**D**,**B**) male (**C**,**D**) and female (**E**,**F**) WT (**left**) and zQ175^Δneo^ (**right**) mice.

Progressive body weight loss is a significant feature in human HD and it has been reported that the original zQ175 mice as well as other HD mouse models showed a steady body weight loss over their lifespan [48,61,62]. To investigate the weight characteristics in our newly generated zQ175^Δneo^ mice as an easy-to-assess biomarker, the body weight was measured on a weekly basis. Male zQ175^Δneo^ mice showed a significant difference of body weight starting at 29 weeks of age. However, the gain of body weight already slowed down at 22 weeks of age, compared to WT littermates (Figure 2A). Female zQ175^Δneo^ also exhibited a significantly reduced body weight as compared to WT littermates, albeit with a slightly later onset (at 32 weeks of age) compared to their male littermates (Figure 2B). We photographed male (Figure 2C,D) and female (Figure 2E,F) 15-week-old (Figure 2C,E) and 57-week-old zQ175^Δneo^ mice (Figure 2D,F). Comparing their body appearance underpins the significant size difference observed in 57-week-old zQ175^Δneo^ mice as compared to healthy, age-matched WT littermates.

**Figure 3:**
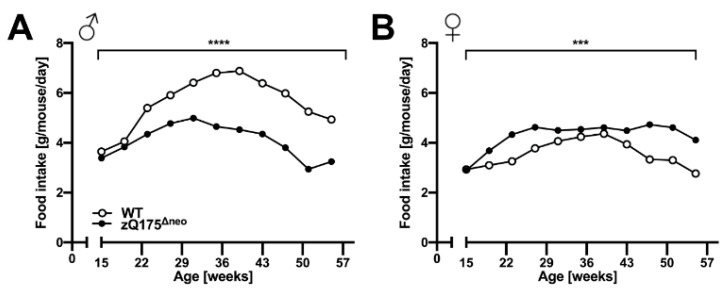
Male (**A**) and female (**B**) zQ175^Δneo^ mice (●) consumed significantly less food compared to WT littermates (○) in the period between 15 and 57 weeks of age. Data are presented as daily consumption per mouse, established from six animals/cage/week. Significance was calculated using paired *t*-tests and is given as ***: *p* ≤ 0.001, and ****: *p* ≤ 0.0001.

In general, multiple factors affect body weight loss making it a complicated metabolic topic in the context of HD [61,62]. However, clinical cases demonstrated that body weight loss is not necessarily the results of the disease itself but rather an insidious consequence of a generally reduced metabolism [63]. Clinically, HD patients usually suffer from swallowing problems and they are more likely to choke, resulting in lower body mass index than healthy individuals [64]. In order to assess the aspect of HD-related body weight loss in zQ175^Δneo^ mice, we also measured the food consumption on a weekly basis. Interestingly, both male and female zQ175^Δneo^ mice consumed significantly less food as compared to their sex-matched WT littermates (Figure 3A,B).

Normal body weight gain and body weight maintenance over time depends on continued sufficient caloric diet and normal physical activity, as well as on the effects of several anabolic functioning hormones and normal hypothalamic function. Hormonal imbalance and impaired hypothalamic function were suggested to contribute to the HD pathogenesis [65,66].

#### 2.1.2. Testicular Atrophy

**Figure 4:**
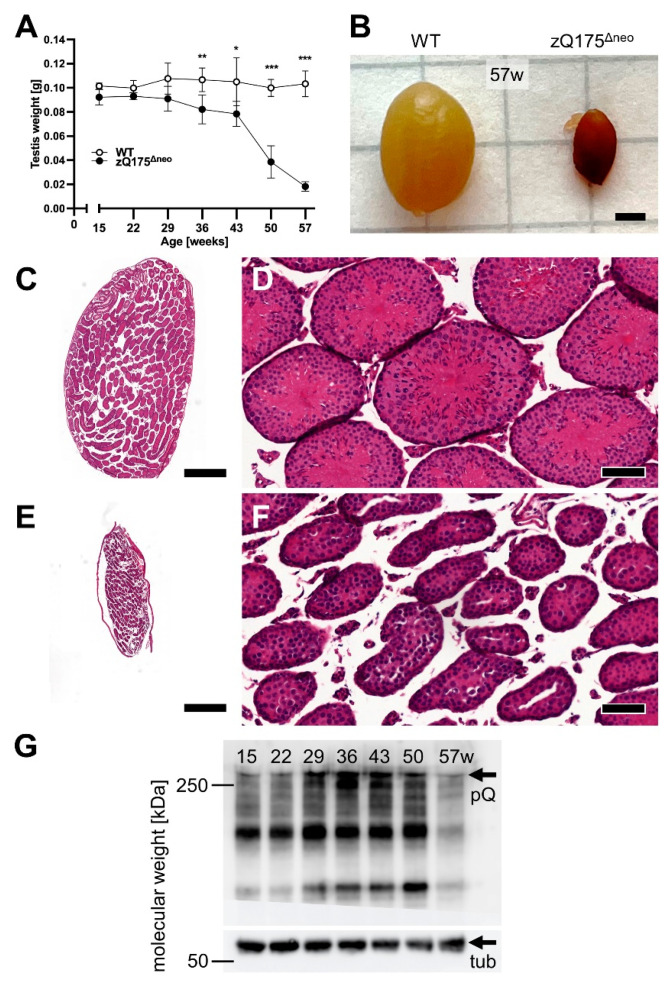
Male zQ175^Δneo^ mice had significantly smaller testicles compared to WT littermates. Testicles of WT and zQ175^Δneo^ mice were collected at 7-week intervals and weighed freshly right after perfusion. (**A**) Testicles’ weight was measured between 15 and 57 weeks of age of male WT (○) and zQ175^Δneo^ (●) mice. Data are presented as mean ± SD; N = 6. Significance was calculated using Mann–Whitney test with Bonferroni-Dunn’s multiple comparisons test and is given as *: *p* ≤ 0.1, **: *p* ≤ 0.01 and ***: *p* ≤ 0.001. (**B**) Representative image showing a direct comparison of testicles of 57-week-old WT (**left**) and zQ175^Δneo^ (**right**). The scale bar indicates 2 mm. (**C**–**F**) Representative images of H&E-stained sagittal testicular sections; overview (**C**,**E**; the scale bar indicates 500 µm) and close-up (**D**,**F**; the scale bar indicates 50 µm) of testicular seminiferous tubes in WT (**C**,**D**) and zQ175^Δneo^ mice (**E**,**F**) at 57 weeks of age. (**G**) Western blot analysis of muHTT [polyQ (pQ) antibody, >>250 kDa] in testicles of zQ175^Δneo^ mice 15–57 weeks of age. muHTT accumulates with increasing age (15–43 weeks) and then declines (50–57 weeks). Highly atrophic, 57-week-old (57w) testis have strongly reduced muHTT. The polyQ antibody (clone MW1) also detects N-terminal Htt fragments. Loading control: tub—β-tubulin, 55 kDa.

Another pathological hallmark of HD is testicular atrophy, which has been observed in male HD patients and male mice [48,67,68]. Thus, we collected testes from male heterozygous zQ175^Δneo^ mice at all experimental time points. Strikingly, significant testicular atrophy was demonstrated from 36 weeks of age (starting at 29 weeks of age at sub-significant levels), as seen by testicular weight loss in zQ175^Δneo^ mice as compared to their WT littermates (Figure 4A). The reduction exceeded 50% of testis weight from 50 weeks of age. Figure 4B shows a representative photograph taken at 57 weeks of age.

Although HD is considered a dysfunction of the CNS and muHTT protein is mainly expressed in the brain, muHTT expression has been detected in the testicles before [48,67]. In fact, human brain and testes share a surprising number of molecular characteristics [e.g., prion (PRP, CD230) and doppel (DPL, PRND) protein expression] and highest common proteins amongst other organs [69]. Thus, we morphologically analyzed the testicles of male zQ175^Δneo^ mice compared to their WT littermates. The analyses revealed a striking massive degeneration of seminiferous tubes in zQ175^Δneo^ mice (Figure 4C–F), and Western blot analysis of muHTT protein demonstrated increased muHTT concentrations over the time period between 15 and 50 weeks (Figure 2G). Testicular atrophy can be caused by different effects: (i) cellular and/or tubular atrophy; (ii) dysfunction of Leydig cells and/or Sertoli cells; (iii) pituitary dysfunction; and (iv) hypothalamic dysfunction. This suggests again hypothalamic dysfunction contributing to HD pathogenesis.

#### 2.1.3. Motoric and Behavioral Changes

Chorea major and other motoric dysfunctions are key features and clinical hallmarks of HD that define the severity of HD [1]. These features have been determined in several HD mouse models before [45,46,70]. To evaluate the motoric impairments and other muscle-related deficits in our model, we conducted accelerating rotarod, wire hang, and pole performance tests at 7-week intervals between 15 and 57 weeks of age.

**Figure 5:**
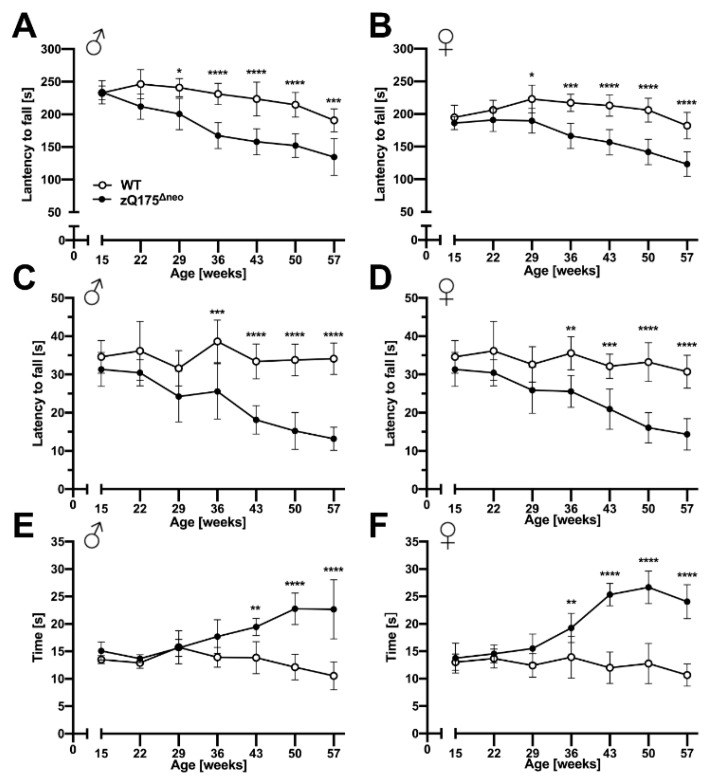
zQ175^Δneo^ mice exhibited robust, progressive motor abnormalities compared to WT mice. The motor performance of male (**A**,**C**,**E**) and female (**B**,**D**,**F**) WT (○) and zQ175^Δneo^ mice (●) was assessed at 7-week intervals by performing accelerating rotarod (**A**,**B**), wire hang (**C**,**D**), and pole (**E**,**F**) performance tests. Data are presented as mean ± SD; N = 4–6. Significance was calculated using two-way ANOVA with Bonferroni’s multiple comparisons test and is given as *: *p* ≤ 0.05, **: *p* ≤ 0.01, ***: *p* ≤ 0.001, and ****: *p* ≤ 0.0001.

Both male and female zQ175^Δneo^ mice displayed a significant decline of their motoric performances in the accelerating rotarod test after 29 weeks of age as measured by decreased time spent on the apparatus before falling (Figure 5A,B). After symptom onset, the rotarod performance declined further over the time course of our experiments. Of note, we observed the decline of rotarod performance consistently on all three days of the test protocol at any week of age (Figure S1).

The wire hang test revealed a similar pattern of reduced forelimb strength of zQ175^Δneo^ compared to their WT littermates. By the age of 36 weeks, both male and female zQ175^Δneo^ mice fell significantly earlier from the wire than their WT littermates (Figure 5C,D). This deficit progressively worsened while the WT mice were stable over the time course between 15 and 57 weeks of age.

Lastly, we used the pole test to assess the basal ganglia-related motor function. We found that male and female zQ175^Δneo^ mice showed normal performance before 29 weeks of age but required significantly more time to turn on top of the pole and subsequently descend it by the age of 43 and 36 weeks, respectively (Figure 5E,F).

Neuropsychiatric symptoms like depression, anxiety, and apathy are common features of HD and they normally manifest prior to motoric deficits [51,71]. Similarly, cognitive decline is a key hallmark of HD that may precede the onset of motor symptoms in HD patients [71]. Thus, we evaluated the behavior of zQ175^Δneo^ mice compared to their WT littermates. Anxiety-like exploratory behaviors were determined by the open field test, in which the main measure is the time spent in the center of an open area. In addition, we assessed other parameters, as total travelled distance and mobility rate as indicators of the general locomotor exploratory activity.

**Figure 6:**
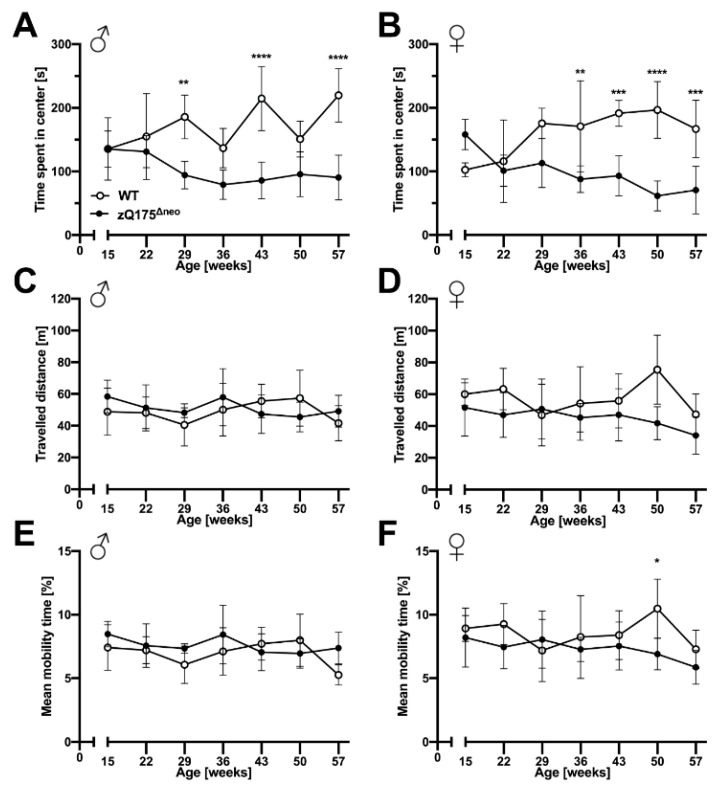
zQ175^Δneo^ mice displayed anxiety-like behavior. The explorative behavior of male (**A**,**C**,**E**) and female (**B**,**D**,**F**) WT (○) and zQ175^Δneo^ mice (●) was evaluated with the open field test assessing the time spent in the center (**A**,**B**), travelled distance (**C**,**D**), as well as mean mobility (**E**,**F**). Data are presented as mean ± SD; N = 4–6. Significance was calculated using two-way ANOVA with Bonferroni’s multiple comparisons test and is given as *: *p* ≤ 0.05, **: *p* ≤ 0.01, ***: *p* ≤ 0.001, and ****: *p* ≤ 0.0001.

We observed that male and female zQ175^Δneo^ mice started to spend less time in the center starting at 29 and 36 weeks of age, respectively (Figure 6A,B). In contrast, we did not observe differences in general exploratory behavior as we found no differences in the total travelled distance and the general mobility rate during the trial period of 10 min (Figure 6C–F). In essence, our results suggest the presence of neuropsychiatric features in zQ175^Δneo^ mice that are found in clinical HD.

#### 2.1.4. Atrophy of Brain Regions

**Figure 7:**
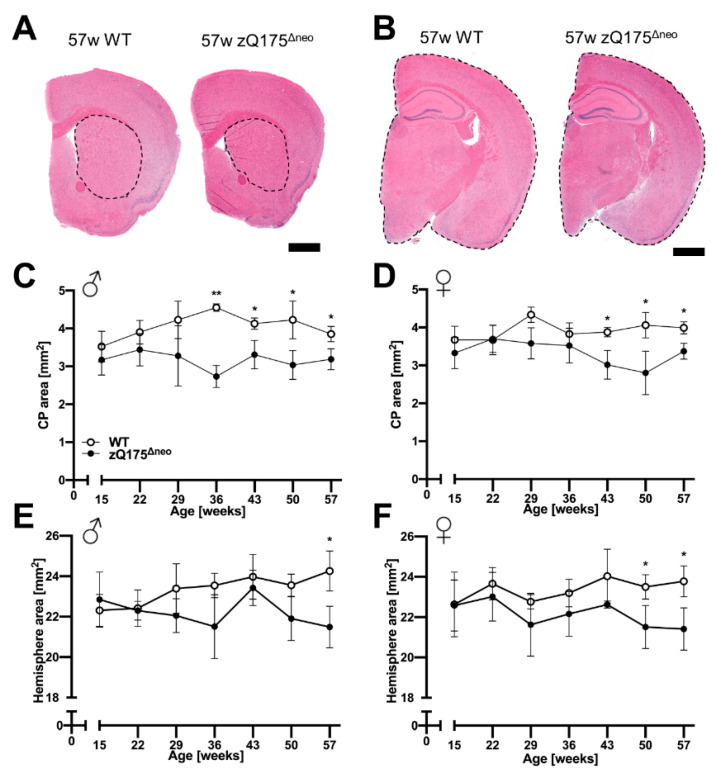
Caudate-putamen (CP) and total hemispheric atrophy were observed in adult zQ175^Δneo^ mice compared to WT littermates. (**A**,**B**) Representative images of H&E-stained coronal brain sections from 57-week-old female WT and zQ175^Δneo^ mice at (**A**) +0.8 mm and (**B**) −1.80 mm distance from bregma. Dashed lines indicate the measured area of (**A**) CP and (**B**) total hemisphere. The scale bars indicate 1000 µm. (**C**–**F**) Male (**C**,**E**) and female (**D**,**F**) zQ175^Δneo^ mice (●) show a reduction of the CP area (**C**,**D**) and the total hemisphere (**E**,**F**) compared to WT littermates (○). Data are presented as mean ± SD; N = 2–6. Significance was calculated using unpaired *t*-test followed by Holm-Šídák correction and is given as *: *p* ≤ 0.05, and **: *p* ≤ 0.01.

Cerebral cortex and striatum are the most vulnerable and affected regions in brains of HD patients [1]. Moreover, atrophy of CP and hemispheres have also been described as neurodegenerative markers in zQ175 mice [46,72]. We measured the area of the CP and the total hemisphere at 7-week intervals in H&E-stained brain sections, +0.8 and −1.8 mm relative to bregma, of mice from 15 to 57 weeks of age. Representative images are presented in Figure 7A,B. The CP areas of both males and females started to appear significantly reduced in zQ175^Δneo^ mice at 36 and 43 weeks of age, respectively, compared to those from WT mice (Figure 7C,D). However, the total hemispheric area was not significantly different in males and females until 50 and 57 weeks of age, respectively, compared to WT littermates (Figure 7E,F). Our results indicate a specific loss of neuronal tissue in the CP as a prominent feature of heterozygous zQ175^Δneo^ mice.

#### 2.1.5. Neuroinflammatory/Glial Reaction

A neuroinflammatory reaction, or simply ‘reactive gliosis’ of astrocytes and microglia, is a common hallmark of neurodegenerative diseases including HD [73,74]. Moreover, we have detected location-specific astrogliosis as the important feature in a mouse model mimicking the preclinical stage of sporadic AD [75]. To investigate its presence in our HD mouse model, we performed post mortem, quantitative immunohistological analyses of the CP and the cerebral cortex.

##### Astrocytes

**Figure 8:**
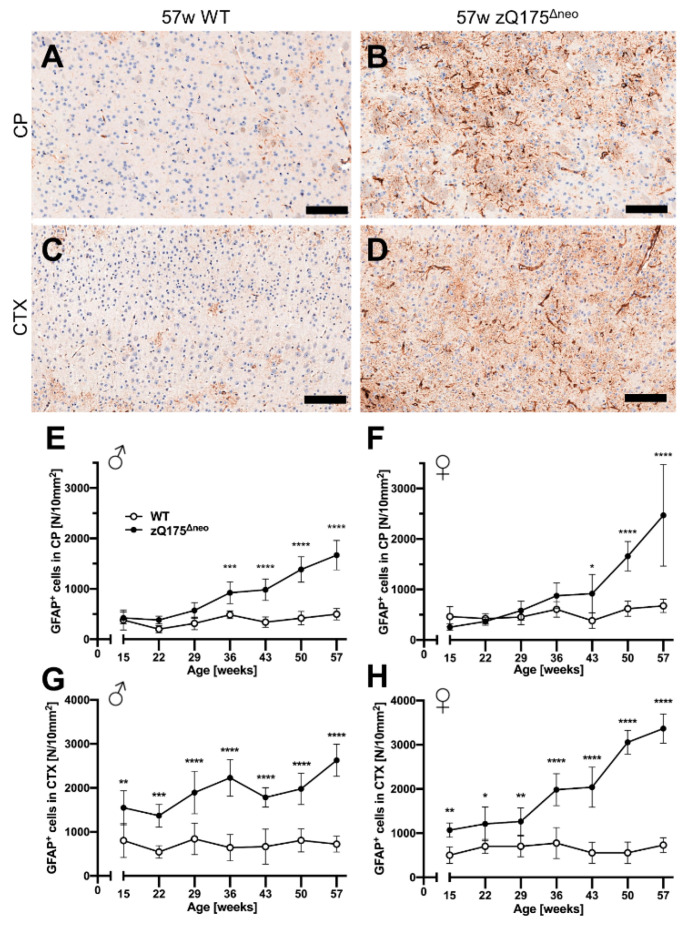
Reactive astrogliosis was observed in the caudate-putamen (CP) from 36 or 43 weeks of age, respectively, and cerebral cortex (CTX) already from 15 weeks of age of zQ175^Δneo^ mice compared to WT littermates. (**A**–**D**) Coronal brain sections from 57-week-old WT and zQ175^Δneo^ mice were immunohistologically stained for GFAP. GFAP^+^ astrocytes were detected using a deep-learning algorithm. Representative images of the GFAP-stained CP (**A**,**B**) and CTX (**C**,**D**) of 57-week-old female WT (**A**,**C**) and zQ175^Δneo^ mice (**B**,**D**). The scale bars indicate 50 µm. Male (**E**,**G**) and female (**F**,**H**) zQ175^Δneo^ mice (●) showed an increase of GFAP^+^ astrocytes compared to WT littermates (○) in both CP (**E**,**F**) and CTX (**G**,**H**). Data are presented as mean ± SD; N = 5–6. Significance was calculated using two-way ANOVA with Bonferroni’s multiple comparisons test and is given as *: *p* ≤ 0.05, **: *p* ≤ 0.01, ***: *p* ≤ 0.001, and ****: *p* ≤ 0.0001.

First, we stained coronal brain sections against the glial fibrillary acidic protein (GFAP), a marker for astrocytes. Representative images are shown in Figure 8A–D. GFAP^+^ astrocytes were detected in specific regions of the whole slide images using a specific deep-learning algorithm as described previously [76,77]. The number of reactive GFAP^+^ astrocytes increased over time in zQ175^Δneo^ mice in the CP (Figure 8E,F) and cerebral cortex (Figure 8G,H), but not in WT littermates. This increase resulted in a significant increase in the CP at 36 or 43 weeks of age, comparing male and female zQ175^Δneo^ mice, respectively, to their WT littermates (Figure 8E,F). However, the increase of GFAP^+^ astrocytes in the cortex was detected already at the first time point analyzed (15 weeks, Figure 8G,H). Summarizing, our results show the robust presence of early reactive astrogliosis and indicate that the first abnormal processes in the brain must have started before the age of 15 weeks in the zQ175^Δneo^ model.

##### Microglia

**Figure 9:**
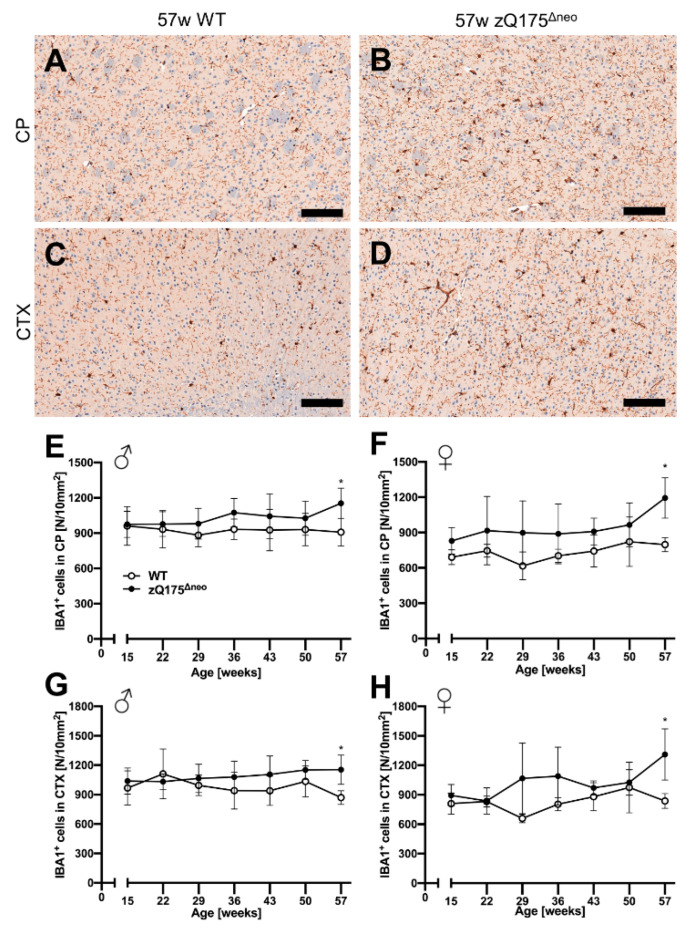
Slight microgliosis was only observed in the caudate-putamen (CP) and cortex (CTX) of zQ175^Δneo^ mice at 57 weeks of age compared to WT littermates. (**A**–**D**) Coronal brain sections from 57-week-old WT and zQ175^Δneo^ mice were immunohistologically stained against IBA1. Representative images of the IBA1-stained CP (**A**,**B**) and CTX (**C**,**D**) of a 57-week-old female WT (**A**,**C**) and a zQ175^Δneo^ mouse (**B**,**D**). The scale bars indicate 50 µm. Male (**E**,**G**) and female (**F**,**H**) zQ175^Δneo^ mice (●) show a significant increase of IBA1-positive microglia as compared to WT littermates (○) in both CP (**E**,**F**) and CTX (**G**,**H**) earliest at 57 weeks of age. Data are presented as mean ± SD; N = 5–6. Significance was calculated using two-way ANOVA with Bonferroni’s multiple comparisons test and is given as *: *p* ≤ 0.05.

Different glial cells are affected and are important contributors to the pathology of HD [78]. Therefore, we further characterized the macrophage-related, neuroinflammatory component of the model by investigating microgliosis in the zQ175^Δneo^ mice. We stained coronal mouse brain sections against ionized calcium-binding adaptor molecule 1 (IBA1) and quantified IBA1^+^ cells with our previously published deep-learning algorithm [76,77]. Representative images are shown in Figure 9A–D. While cortical astrogliosis presented as early as 15 weeks of age (Figure 8), we found the number of microglia in CP and CTX to be similar comparing both male and female zQ175^Δneo^ mice to their respective WT littermates (Figure 9E–H). Only at the latest time point, at 57 weeks of age, zQ175^Δneo^ mice had a significant increase in the number of IBA1^+^ microglia across both sexes and regions.

### 2.2. Charting the Bioactivity Network of Known HD-Targeting Agents

#### 2.2.1. Compilation of the Huntingtin’s Disease Multitarget Dataset (HD_MDS)

**Figure 10:**
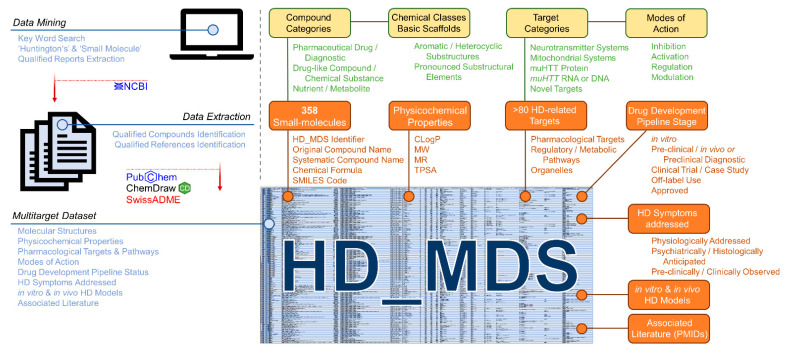
Schematic representation of the establishment of the Huntington’s Disease Multitarget Dataset (HD_MDS, Supplementary Material Table S1).

Rodent disease models and their detailed characterization for several morphological and molecular features over a long period is highly important for the development of new treatment. However, data awareness in terms of pharmacological HD drug targets and potential agents is also crucial for novel drug discovery, design, and development. In fact, the target landscape with respect to HD is rich and diverse, which leaves much space for rational drug design approaches [2,14,15,17,20,21,22,27,35,79]. Many compounds with affinities to one or several of these targets have also been identified. However, there exists no useful database or dataset that correlates the molecular structures of these bioactive molecules to their HD drug targets. Such a database would strongly support both an understanding of interconnected molecular mechanisms in HD pathogenesis and the design of a new generation of bioactive agents against this neurodegenerative disease. Thus, we were prompted to summarize the entirety of small-molecules addressing pharmacological targets associated with HD in a ‘multitarget dataset’ (HD_MDS; Figure 10, Table S1) akin to our previously published ABC_BPMDS [80]. The HD_MDS was accomplished by a data mining approach using the database of the National Center for Biotechnological Information (NCBI) searching for qualified reports applying the key words ‘small-molecule’ and ‘Huntington’s’. The respective qualified reports were also investigated for qualified references. Qualified compounds were listed if they (i) showed positive effects in in vitro HD models; (ii) demonstrated positive outcomes in in vivo HD models; (iii) have been assessed in clinical trials or case studies with participation of human HD patients; or (iv) were designed for diagnostic purposes (e.g., development of PET tracers [54,55,57,81,82,83]) Molecular structures of compounds were retrieved either from the PubChem database or were manually drawn in ChemDraw using provided molecular structure templates from the respective reports.

In total, we identified 358 unique HD-targeting small molecules. These molecules are described with (i) an unique identifier (HD_MDS_ID); (ii) original compound name according to the original report(s) including alternative name if applicable; (iii) systematic compound name according to the IUPAC nomenclature; (iv) chemical formula; (v) SMILES code; (vi) compound categorization [(a) pharmaceutical drug/diagnostic; (b) drug-like compound/chemical substance; (c) nutrient/metabolite]; and (vii) chemical class including basic scaffolds (e.g., pyridine, quinazoline, etc.). Particularly the given chemical classes (e.g., aromatic and heterocyclic substructures or other pronounced substructural elements) will facilitate an optimized search for desired compound classes.

Additionally, determinants that conserve physicochemical features of these bioactive molecules were included, particularly the calculated octanol/water partition coefficient (CLogP), molecular weight (MW), molar refractivity (MR), or the topological polar surface area (TPSA) [80,84]. These parameters were calculated for each compound using the web service SwissADME [85]. Cerebral penetration is key for effective treatment of neurodegenerative disorders such as HD, and these pharmacokinetic determinants have already been identified to crucially establish a correlation between molecular structure of drugs and their interaction with cerebral drug targets [80,84].

The HD_MDS is freely available as Excel-table on the ‘ABCHD’ project resources web page https://doi.org/10.17605/OSF.IO/EJVWY and the www.panabc.info web page (‘Version_1’). Subsequently, it will be complemented and improved to support further drug discovery approaches.

#### 2.2.2. HD_MDS Analysis and Validation—The HD Target Landscape

The compilation of the HD_MDS by data mining revealed that the target landscape in HD is rich and diverse. Over 80 pharmacological targets and pathways were identified in association with the 358 listed HD-targeting small-molecules. In principle, five target categories can be differentiated:

(i) Neurotransmitter systems, which are addressed by many off-label-use anticholinergics, antidepressants, and antipsychotics such as risperidone [14,15,17,19,35,86] mirtazapine [2,14,17,19], and memantine [14,19,20,79,86,87,88]. The respective targets include DRs, 5HTRs, or NMDARs, but also choline esterases/receptors (ChEs/ChRs) [14,15,17,19,35,79,86,87], adrenoreceptors (αRs) [2], and γ-amino butyric acid receptors (GABARs) [14,17,79,87,89]. It shall be mentioned that repurposing of drugs that are already approved for related neurodegenerative diseases is very common in HD treatment [15,17,19,79]. Many drugs listed in the HD_MDS that are either under clinical evaluation against HD or in off-label use to address HD symptoms that overlap with other neurological diseases such as Alzheimer’s disease (AD). Of note, targeting neurotransmitter systems represents to this date purely symptomatic treatment of HD and has in the vast majority of cases not or only very modestly resulted in a benefit in clinical trials and case studies [15,17,86].

(ii) Mitochondrial systems, which are addressed by a large number of molecules, including compounds that protect mitochondria and rescue mitochondrial membrane potential [56,79,86,90,91,92], promote mitochondrial biogenesis [14,15,20,79], as well as enhance mitochondrial respiration and function [14,20,79]. These observations have been made for several compounds, such as minocycline [86,90], fenofibrate [14,15,20,79], and triheptanoin [14,20,79]. As mitochondrial deterioration is assumed to be a side effect of HD, these and other molecules with similar function will most likely not lead to a curative treatment.

(iii) *muHTT* RNA [15,16,30,79,93,94,95,96,97,98,99] or DNA [100], which can be targeted by small-molecules to prevent or modulate transcription and splicing [15,30,79,93] as well as interrupt translation and posttranslational modification [16,94,95,96,97,98] of muHTT. Compounds allocated within this target category include branaplam [15,93], posiphen [16,94], or furamidine [95], thus inhibiting muHTT production. If successful, these interventions could be potentially curative.

(iv) muHTT itself [14,15,35,54,55,57,81,86,101,102,103,104], which can either be subjected to degradation or prevented to aggregate. This was demonstrated, for example, by PBT2 [14,15,35,86] and certain proteolysis-targeting chimeras (PROTACs) [101,102,103]. Here also, if successful, these interventions could be potentially curative.

(v) Novel, diverse targets reducing muHTT production, enhancing muHTT degradation, and generally lowering muHTT-conferred (long-term) toxicity, and thus, positively affect HD onset and progression. These targets include CASPs [2,14,15,17,20,21,22,23,24,25,26,79], HSPs [2,22,27,28,29,30,31,32,33,34], HDACs [2,14,15,17,20,79,105], PDEs [14,15,17,31,35,36], and σRs [14,15,35,37,79], but also the adenosine 2A receptor (A2AR) [14,36], the AMP-activated protein kinase (AMPK) [20,31,79], the ERG-associated protein with SET domain (ESET) [106,107,108], the Kelch-like ECH-associated protein (KEAP) [31,56,109,110], the nuclear factor erythroid 2-related factor 2 (NRF2) [20,31,109,110], the peroxisome proliferator-activated receptor γ coactivator 1α (PPARGC1A) [14,15,20,35,79], the protein disulfide isomerase (PDI) [111,112], the protein kinase A (PKA) [31,36], the Rho-associated protein kinase (ROCK) [36,113], and sirtuins (SIRTs) [14,20,35,86,114].

These targets are frequently addressed by small-molecules, even though the detailed mechanisms of action have yet to be elucidated in most of the cases. Nevertheless, these novel drug targets open up the possibility for potentially curative treatments against HD. It should be noted that for some compounds, more than one target category is eligible. However, a large portion of compounds of the HD_MDS cannot be allocated to one of these groups due to insufficient data on the background of their mode of action (marked with ‘Target Category Unknown’).

#### 2.2.3. HD_MDS Analysis and Validation—The HD Polypharmacology Landscape

The HD_MDS allows not only for the discovery of different molecular-structural classes of drugs, drug-like compounds, chemical substances, nutrients, and metabolites to address one particular (potentially novel) target. It enables also for the discovery of agents that address several pharmacological (potentially novel) targets. This bioactivity network is crucial for the understanding of HD and the development of novel therapies, and thus, one important outcome of the HD_MDS.

In fact, most drugs of the HD_MDS currently used to treat HD by addressing neurotransmitter systems are actually polypharmaceutics that address several target structures. Particularly the agonistic or antagonictic interplay or secondary effects on adrenergic, cholinergic, dopaminergic, GABA-ergic, and serotoninergic receptors is the basis for their very pharmacological effect that may ameliorate HD symptoms under certain circumstances [14,15,17,86,87,89,115].

**Figure 11:**
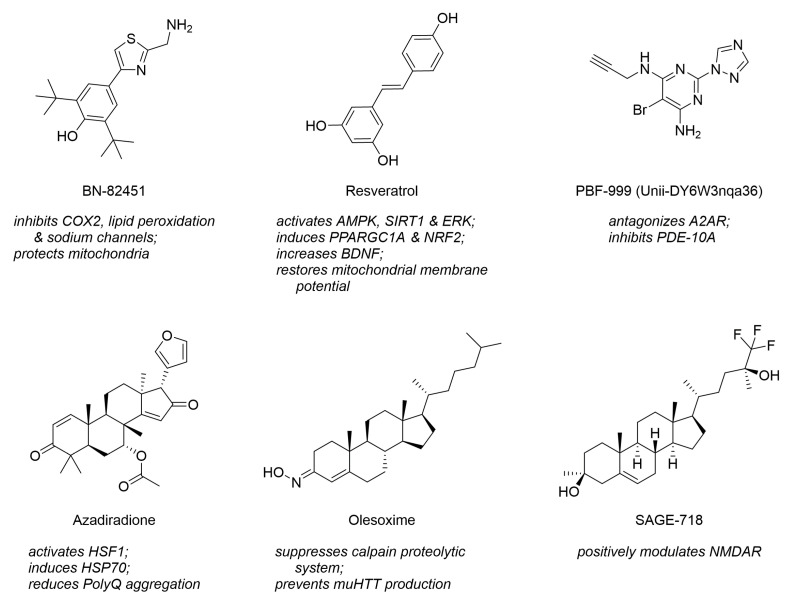
Molecular formulae of polypharmaceutics and polypharmacological compound classes that stretch their biological activity over a range of diverse HD-related pharmacological targets and pathways.

Interestingly, many novel drug-like compounds in preclinical evaluation address also more than one of the novel, diverse drug targets. For example, the thiazole derivative BN-82451 was demonstrated to be a polypharmacological inhibitor of cyclooxygenase 2 (COX2) and voltage-gated sodium ion channels (VGSCs) [14,116]; the stilbenoid resveratrol showed to address AMPK, PPARGC1A, and SIRT [20,35,79]; and the pyrimidine derivative PBF-999 addressed PDE-10A and A2AR. Figure 11 visualizes these molecules. Furthermore, polypharmacology is not only a feature of individual molecules but also entire compound classes. Very prominent examples are steranes or sterane-like molecules, such as azadiradione [28], beclomethasone [39], betamethasone [39], budesonide [39], carbenoxolone [117], celastrol [38], deacetoxy-7-oxogedunin [29], deacetylgedunin [29], deoxygedunin [29], desonide [118], 18β-glycyrrhetinic acid [117], gugglesterone [39], hydrocortisone [39] olesoxime [16,119], ouabain [117], prednisolone [39], proscillaridin A [117], triamcinolone [39], ursodeoxycholic acid (ursodiol) [14], and withaferin A [120]. These address several target categories, such as mitochondrial systems and novel, diverse targets. Figure 11 depicts three representatives of steranes that address individually diverse HD-related pharmacological targets. Steranes and other compound classes stretching over target space pose a new opportunity for future HD-targeting drug design and development.

## 3. Discussion

### 3.1. Interpretation of the New In Vivo Assessment Platform

Previously, zQ175 mice have been used as a promising HD mouse line with a subtle but significant onset and progression of HD in both the heterozygous and homozygous state [45,46].

In the present study, we performed a series of behavioral and histopathological experiments with a new zQ175^Δneo^ mouse line and their WT littermates at various points in time (15, 22, 29, 36, 43, 50, and 57 weeks, respectively) to thoroughly characterize the time course of HD-induced behavioral and neuropathological changes. Our new, *neo*-excised zQ175^Δneo^ model exhibited long-term phenotypes starting with symptoms from an early age of the mice lifespan. Specifically, zQ175^Δneo^ showed early growth retardation, body weight loss, and anxiety-like behaviors latest at 29 weeks of age, prior to most motor deficits, such as motor balance, coordination, or muscular strength, which manifested earliest at 36 weeks of age. These findings are in contrast to some N-terminal fragment HD models, e.g., R6/2 mice, which usually have a shorter lifespan and an earlier onset of most HD symptoms [121]. In this regard, zQ175^Δneo^ mice have a great in vivo potential for exploring preventative interventions of HD.

One of the original characterization studies of zQ175 mice reported apparent weight loss from 3 months in the heterozygous mice and 1.5 months of age in the homozygous, respectively [45]. The onset of this essential HD symptom was delayed after the deletion of the *neo* cassette, resulting in a significant statistical body weight segregation from their WT littermates earliest at 29 weeks in males and at 32 weeks of age in females. Growth retardation became already visible at 22 weeks of age. This pronounced body weight loss was accompanied by a progressive decrease of food intake within the entire study period (15–57 weeks). Given the clinical fact that the severity and onset age of HD are often associated with the length of CAG repeats, these zQ175^Δneo^ mice showed still a much earlier weight loss than a comparable KI HD model [122]. HdhQ200 mice carry a similar number of CAG repeats (~200) and only show an obvious weight loss at 50 weeks of age in heterozygous individuals [122]. These findings suggest that the onset age of body weight loss, at least in HD mice, is not directly linked to the CAG expansion length per se. Instead, the deletion of the *neo* cassette, the genetic background, the effects of muHTT expression in testis, and the specific genetic constructs are important regulators of the overall disease course and symptom onset. These aspects were directly assessed and described in the present study. Of note, some transgenic HD mouse models such as BACHD and YAC128 mice displayed even a body weight increase during the disease progression [42,43]. However, these rather rare observations contradict the majority of findings [48,61,62] and could be a confounding factor to motor function tests [43,123]. In essence, the new zQ175^Δneo^ mice represent an appropriate mouse model for studying HD-related metabolic abnormities manifesting an early, reproducible, and well-defined body weight loss, recapitulating human HD. Importantly, the high reproducibility of the growth pattern defines the optimal treatment window for drug screening, e.g., start for assessing preventive treatments at 15 weeks for zQ175^Δneo^ mice. Body weight assessment has been described before as a robust and easy to perform, non-invasive measure in HD [124].

Along with the body weight loss, we also found significant testicular atrophy after 36 weeks of age in zQ175^Δneo^ mice. Testicular atrophy and expression of muHTT in the testicles is an important hallmark of HD [48,67,68]. In contrast, testicular atrophy was not detected in zQ175 (C57BL/6J background) but Q175 and Q175FDN mice (both in FVB/N background) [48]. Our results indicate that this adult-onset phenotype may be associated with the deletion of neomycin selection cassette and the resulting enhancement of muHTT toxicity. Additionally, our histological analysis of the testicles revealed degenerated seminipherous tubules throughout the 57 weeks of the time course, which is an important corresponding feature of human HD symptoms [67,125].

Motor dysfunction is an important feature when assessing HD mouse models. Several studies reported that motor abnormalities have been recapitulated in different HD mouse models [45,46,70]. In our work, we employed a series of behavioral tests including accelerating rotarod, wire hang, and pole performance tests to determine the time course of motor function decline throughout the course of the disease. Our results revealed an age-related, progressive pattern of motor deficits in zQ175^Δneo^ mice. The motor coordination deficits initiated as early as 29 weeks of age in both males and females. This is comparable to the original zQ175 mouse model, in which both homozygous and heterozygous mice display deficits in the rotarod performance test at an age of 30 weeks.

Cognitive decline is a key hallmark of HD. Several studies have shown that cognitive deficits can even precede the onset of motor symptoms in patients [71], which can cause diagnostic problems for patients with de novo CAG expansions. Using the open field test, we found that zQ175^Δneo^ mice spent less time in the center of the arena as early as 29 and 36 weeks of age in males and females, respectively. The absence of exploratory deficits, particularly the travelled distance and mobility rate in the open field test, indicates that this is not caused by muscular dysfunction and problems with locomotor activities. Menalled et al. trained zQ175 mice to perform a simple procedural response learning. They found significant learning deficits only at 58 weeks of age [45]. The interpretation of these results can be difficult since the advanced age is a least a co-factor interfering with learning abilities in these mice.

Cerebral cortex and striatum are the most vulnerable and affected regions in HD brains [1]. We found general atrophy of CP and hemispheres in the present study, which is consistent with human HD and other HD mouse models, such as R6/2 [126]. Notably, the CP showed earlier onset and greater extent of atrophy in zQ175^Δneo^ mice compared to the total hemispheres. These results resemble human HD, in which the striatum is the most degenerated region with extensive death of MSNs [127]. We found a reduction of the hemisphere cross sections only at 57 weeks of age, the end of our study. A study of R6/2 mice reported whole brain atrophy using magnetic resonance imaging (MRI) already at 4 weeks, which was progressing with age [128]. In another study, MRI investigation of heterozygous zQ175 mice revealed significant atrophy affecting the cortex and CP at 4 months of age [129]. These studies suggest using a more advanced method such as MRI to assess regional and global brain atrophy in future.

Astrogliosis and microgliosis are neuroinflammatory processes that react on or contribute to HD pathology [73,74,78], which prompted us to characterize neuroinflammation in heterozygous zQ175^Δneo^ mice. Consistent with previous reports of HD patients and mouse models, we observed HD-induced astrocytosis at 36 weeks of age in the CTX but already at 15 weeks of age in the CP of zQ175^Δneo^ mice. This early and robust astroglial response in the CP persisted throughout the whole time course of the study and reached a five-fold increase at the final time point compared to WT littermates. Our findings imply that the phenotypic changes of zQ175^Δneo^ mice are not immediately correlated with the activation of astrocytes neither in the CTX nor in the CP, which was supported by the literature [130]. Instead, the classic astrogliosis occurred much earlier than symptomatic manifestations. Several studies have found that the selective expression of muHTT in astrocytes results in striatal neurodegeneration [131], suggesting astrocytes as a preventative therapeutic target for HD. Various other studies have reported remarkable changes of astrocytes in HD mouse models, e.g., altered transcriptional profiles in R6/2 mice at 8 weeks of age [132], reduced GFAP in R6/2 mice [130], or HD-induced reactive astrogliosis in zQ175 mice at 6 months of age [133].

In the context of HD, microglia changed the morphology with an increased soma size in the early stage of HD progression [134]. These morphological changes occurred even without changes in the number of microglia [135]. Moreover, microglia in HD mice exhibited an upregulation of pro-inflammatory cytokines. For example, at the end of disease progression, interleukin (IL) 6, IL-10, and IL-12 are all increased in the CP of zQ175, YAC128, and R6/2 HD mice [136,137]. In line with these findings, we observed elevated numbers of activated microglia in the CP and CTX, but only at the final age of our characterization at 57 weeks of age. Thus, we can state that early microglial activation may not be a necessary key feature for the onset of HD pathogenesis.

In summary, the zQ175 model was the first heterozygous KI mouse strain that exhibited an apparent and early HD-like phenotype [45]. In an attempt to enhance the HD-like symptoms for preclinical use, the neomycin resistance cassette was excised from the *Htt* gene locus of zQ175 mice, generating a new line, zQ175^Δneo^. We described the time course of behavioral, neuropathological, and immunohistological changes of zQ175^Δneo^ mice, which better resemble the human situation and present with early, robust behavioral and cellular alterations in heterozygous mice compared to the existing HD mouse models.

### 3.2. Interpretation of the HD_MDS

In addition to the development of an improved, better suitable, and comprehensively assessed in vivo HD mouse model, we have, for the first time, provided an extensive dataset correlating the molecular structures of molecules to a rich and diverse set of (potential) HD drug targets, and thus, elucidated the bioactivity network of HD-addressing small-molecule agents. The HD_MDS revealed that there is a considerable but still barely charted target space beyond ‘usual’ pharmacological HD-related targets. Strikingly, through uncovering the bioactivity network, two major opportunities occur: (i) identification of recurring protein (super-)families that stretch through the entire dataset and are addressed by several, structurally different molecules; and (ii) identification of recurring molecular scaffolds that simultaneously address several structurally and functionally different target proteins.

#### 3.2.1. Recurring Targets: Solute Carriers and Other Transporters

The superfamily of solute carrier (SLC) transporters is prominently represented within the target space of the HD_MDS. Several compounds targeting HD and other neurodegenerative diseases address SLC transporters, such as atomoxetine [norepinephrine transporter (NET), SLC6A2] [14,17,138], bupropion [NET and dopamine transporter (DAT), SLC6A3] [14,35], fluoxetine [serotonin transporter (5-HTT) alias serotonin transporter (SERT), SLC6A4] [14,15,17,20,86,139], riluzole [glutamate transporter 1 (GLT-1) alias excitatory amino acid transporter 2 (EAAT2), SLC1A2] [20,140], tetrabenazine [vesicular monoamine transporter 2 (VMAT2); SLC18A2] [14,15,17,19,20,79,86], or biotin [thiamin transporter 2 (ThTr2), SLC19A3] [141].

This may not come as a surprise as SLC transporters are crucially involved in cerebral neurotransmitter distribution. However, many more SLC transporters, particularly their dysfunction and dysregulation, were found to be associated with HD. EAAT1 (*SLC1A3*), for example, was found to be upregulated in humans [142]. The closely related EAAT3 (*SLC1A1*) was downregulated in one mouse model of HD [143] but unchanged in another [144] and upregulated in a cell-based model [145]. Various other, mostly vesicular neurotransmitter transporters were also associated with HD, including vesicular acetylcholine transporter (VAChT, *SLC18A3*) [146,147], vesicular glutamate transporter 1 (VGluT1, *SLC17A7*) [142], VGluT2 (*SLC17A6*) [148], and vesicular inhibitory amino acid transporter (VIAAT, *SLC32A1*) [149]. The potassium-chloride co-transporter 2 (KCC2, *SLC12A5*) [150] and sodium-potassium-chloride co-transporter 1 (NKCC1, *SLC12A2*) [150,151], which modulate the GABA-ergic system, were found to be down- and upregulated, respectively. The SLC2A family of glucose transporters was also strongly implicated in HD, with GLUT1 (*SLC2A1*) and GLUT3 (*SLC2A3*) expression shown to be 3- and 4-fold decreased in late stage patients [152], while an increase in GLUT4 (*SLC2A4*) expression was seen in a different study [153]. Other SLCs with increased expression in humans in association with HD were divalent metal transporter 1 (DMT1, *SLC11A2*; although not statistically significant) [154], equilibrative nucleoside transporter 1 (ENT1, *SLC29A1*) [155], monocarboxylate transporter 9 (MCT9, *SLC16A9*) [142], prostein (*SLC45A3*) [156], and urea transporter 1 (UT1, *SLC14A1*) [142]. Other SLCs that have been associated with HD in different ways are bicarbonate transporter-related protein 1 (BTR1, *SLC4A11*) [157], sodium-hydrogen exchanger 1 (NHE1, *SLC9A1*) [158], organic cation/carnitine transporter 2 (OCTN2, *SLC22A5*) [159], phosphate carrier protein (PHC, *SLC25A3*) [160], *SLC3A2* [160], sodium-dependent vitamin c transporter 2 (SVCT2, *SLC23A2*) [161], and zinc transporter 10 (ZnT10, *SLC30A10*) [162].

The involvement of SLC transporters raises immediately the questions if altered metabolite and drug distribution is a hallmark of HD and even other, phylogenetically unrelated membrane transporter families, e.g., (vesicular [163]) ATP-binding cassette (ABC) transporters, participate in the pathogenesis of HD. Various ABC transporters have been associated with neurodegenerative diseases, e.g., AD, Lewy-body dementia, and Parkinson’s disease [84,164,165,166,167,168,169,170,171] and are expressed in specific structures of the brain [172,173]. The drug transporters ABCB1, ABCC1, and ABCG2 were demonstrated to directly clear amyloid-β (Aβ) and to be dysfunctional in AD brains [174,175]. Furthermore, the lipid transporters ABCA1 and ABCA7 were shown to affect Aβ production, degradation, and clearance through yet unknown mechanisms [165,176,177,178]. Given the neurotoxic effects of Aβ as well as muHTT and other poly-glutamine deposits, and considering the neuroprotective nature of ABC transporters, a contribution of these transporters to the pathogenesis of HD should be investigated. ABCA1, for example, is an integral part of the homeostasis and metabolism pathways of cholesterol. Dysfunctional cholesterol homeostasis as a result of either (i) the lack of interaction of intrinsic and functional HTT [179], or (ii) the interaction of muHTT [180,181] with different components of the cholesterol metabolism pathway is a hallmark of HD-affected neurons [180,181]. Two important regulators of ABCA1 [165], which were found to be downregulated in cellular HD models [179], are the liver-x-receptor α (LXR-α) [179] and the sterol regulation element-binding protein 2 (SREBP2) [180,181]. These interactions lead to a reduced cholesterol biosynthesis, particularly in astrocytes [180,181]. The lack of cerebral cholesterol may lead to a reduced expression of other ABCA transporters, such as the phospholipid and cholesterol transporter ABCA7 [165]. The cholesterol transporters ABCG1 and ABCG4 were already associated with dysfunctional cholesterol homeostasis and HD [182,183].

Apart from cholesterol transporters, other ABC transporters were associated with neurodegenerative diseases [167,169,184]. ABCB10 is involved in the mitochondrial unfolded protein response (UPRmt) pathway [185], which is downregulated in murine HD striatal cells, fibroblasts derived from HD patients, and R6/2 mice [186,187,188]. Deletion of ABCB10 caused increased mitochondrial reactive oxygen species (ROS) and cell death, whereas overexpression of ABCB10 reduces these effects [186]. Similar events were also described for peroxisomal ABCD1, a fatty acid transporter [189]. Decreased glutathione (GSH) transport was observed by muHTT-mediated downregulation of the multidrug transporter ABCC1 [190]. Multidrug transporters of the ABCC sub-class were further discussed in terms of their impact on CNS-penetration and clearance of potential HD drugs [99].

Considering the positive effects of steranes and sterane-like molecules on HD pathology in various HD models [14,16,28,29,38,39,117,118,119,120], particularly the cholesterol (and phospholipid) transporters ABCA1 (and potentially its functional compensatory counterpart ABCA7 [164,165]) gain relevance in the pathogenesis of HD and potential therapeutic and diagnostic interventions, which warrants further investigations.

#### 3.2.2. Recurring Scaffolds: Perspective on Future HD Drug Discovery and Development

Another crucial discovery of the in-depth analysis of the HD_MDS is that particularly novel, diverse HD drug targets are frequently addressed by structurally and functionally novel small-molecules. These associations demonstrate the interconnectivity of these targets and may even hint to the existence of novel, yet undiscovered targets and pathways in the pathogenesis of HD. The polypharmacological nature of many HD-targeting compounds may be the primary reason for their effectiveness, e.g., in case of neurotransmitter systems-targeting agents. A polypharmacological approach by intentional design of multitarget agents could revolutionize HD drug development by uncovering molecular-structural dependencies of novel, yet undiscovered pharmacological targets and modes of action.

We have recently developed a computational methodology called ‘computer-aided pattern analysis’ (‘C@PA’) which was particularly designed for the generation of polypharmaceutics [191,192,193]. This methodology uses molecular-structural patterns derived from a multitarget dataset of ABC transporter inhibitors for the prediction of novel, structurally distinctive bioactive agents by screening of chemical space. Transferring this methodology to the HD_MDS may result in new, structurally distinctive, and functionally novel molecules with advanced modes of action and optimized polypharmacological profiles that establish a new generation of preclinical and clinical HD-targeting agents.

### 3.3. Conclusion, Outlook, and Hypotheses

Using the new and fully characterized zQ175^Δneo^ mouse model, we can suggest different strategies to assess treatment effects of new compounds. The detailed characterization of phenotypical and morphological features pinpoints towards the use of different treatment paradigms for the drug screening, in particular (i) preventive treatment before onset of symptoms; (ii) post-onset treatment; and (iii) late treatment after onset of several phenotypical symptoms.

(i) Having detected early astroglial activation already at 15 weeks of age without further phenotypical abnormalities, we propose this time point as optimal start for preventive treatment screens with an assessment horizon beyond 30 weeks of age. The assessment parameter that could be used in the ongoing screening is the ‘prevention of growth retardation’. Growth retardation starts at 22 weeks of age as a non-significant change, leading to a significant regression in body weight from 30 weeks of age in male mice compared to WT littermates (Figure 2). This paradigm represents best the situation of inherited forms of HD.

(ii) The sole effect of bodyweight recovery after a treatment start at 29 weeks of age could be used as early post-onset evaluation, representing best the sporadic patients’ situation. Here, the analysis horizon should be at least 14 weeks (43 weeks of age).

(iii) To screen for new drugs modifying disease pathogenesis, the late-treatment paradigm appears less practicable due to already exciting loss of neurons and cemented phenotypes.

Easy to assess, minor interfering, and robust parameters that can be assessed repeatedly should be preferred over complicated assessments. For the zQ175^Δneo^ mice and the preventive paradigm, we suggest initial motoric assessment using the rotarod performance test at the treatment starting point of 15 weeks in groups of 15–20 mice, weekly weight and food intake measurements, re-assessment of motoric skills at 29 weeks, and a total treatment time up to 36 weeks of age with a final motoric re-assessment. Depending on the effect of the chosen drug and application (daily gavage, in drinking water, etc.), the total treatment time can be extended to 43 or even 50 weeks of age. Continuously measured weight curves are an easy tool to determine the effect onset and effect size.

Besides using better experimental strategies and well-characterized animal models, new and innovative drug targets have to be determined. During the past years, several members of the ABCA transporter subfamily have been linked to neurodegenerative diseases [164,165,194,195]. The ABCA subfamily is known to transport a variety of lipid-soluble substances and metabolites, amongst them the sterane cholesterol, phospholipids, and retinol [165]. These agents could be the link why the APOE allele ε4 is the strongest genetic risk factor for sporadic AD [165]. In HD, members of the ABCA transporter family have not yet been identified in genome wide association studies (GWASs) [196,197,198]. This is not surprising since it took many years and GWASs as well as genetic variant studies to identify ABCA1 and ABCA7 as AD risk genes despite more than two decades of experimental evidence [165,195,199,200,201,202,203].

We propose that members of the ABCA subfamily are involved in disease modification or even pathogenesis of HD. Many molecules similar to cholesterol have demonstrated positive effects on muHTT production, toxicity, and degradation without knowing their particular mode of action [38,39,117,118]. Are ABCA transporters the missing link between sterane(-like) molecules and positive treatment outcomes? The testis atrophy detected in some HD mice and patients due to low production of the sterane testosterone caused by testicular muHTT accumulation or as secondary deficit due to problems of the pituitary-hypothalamic driver axis puts forward the question whether cholesterol transporters are involved herein as well. Do ABCA transporters play a role in regulating the hypothalamic or testicular function and does pathological muHTT accumulation directly or indirectly influence ABCA transporters’ expression in these important regulatory brain regions? We propose that cholesterol transporters, such as ABCA subfamily members, influence important metabolic regulators in the basal ganglia and hypothalamus and should be investigated in more detail.

## 4. Material and Methods

### 4.1. Animal Models and Breeding Scheme

Subjects were male and female heterozygous zQ175^Δneo^ mice and their corresponding WT littermates generated from the original C57BL/6JQ175KI HD (zQ175) mouse model [45]. To delete the neomycin selection cassette in the HD mice, we mated male zQ175 mice (189Q, Table S2) [44] (JAX stock #027410) with female CMV-*cre* mice (JAX stock #006054), which express ubiquitous Cre-recombinase (also in germ cells) and were used to excise the neomycin cassette (Figure 1) [49]. Afterwards, the CMV-*cre* transgene was negatively selected and taken out from the background. The mouse colony was maintained in the C57BL/6J background. Experimental mice were housed in groups of six animals per cage with free access to chow food and autoclaved acidified (pH 3) water. Mice were housed at the animal care facility of the Department of Comparative Medicine (KPM, Radiumhospitalet, Oslo, Norway). All animals were maintained under standard conditions of temperature (22 °C), relative humidity (62%) and an artificial dark-light cycle of 12 h/12 h in the facility. Mice were genotyped after weaning and heterozygous zQ175^Δneo^ mice were identified by polymerase chain reaction (PCR) of DNA extracted from ear biopsies (for primer details see Table S2).

### 4.2. Body Weight and Food Consumption

Individual body weight of mice was monitored continuously (weekly measurements, 15 to 57 weeks of age). Food consumption was measured as g/week per cage (with six mice each).

### 4.3. Experimental Design

In this study, zQ175^Δneo^ mice and WT mice were evaluated at seven distinct time points during disease progression: 15, 22, 29, 36, 43, 50, and 57 weeks of age, respectively. These time points were chosen to characterize the behavioral and neuropathological characteristics of this HD mouse model at seven different stages of the lifespan. To eliminate any possible confounding influence in mouse behavioral tests due to repeated testing, seven distinct cohorts of animals were set up. Every cohort consisted of sex-matched zQ175^Δneo^ mice and their WT counterparts (N = 6 per genotype and sex; N may differ for each test, see Table S3 for details). All behavioral tests were performed during the light phase of the dark/light cycle. Mice were transferred to the experimental room together with their home cages and acclimated for at least one hour before the start of the experiments. The experimenters were blinded to the genotypes at the time of behavioral testing where possible. Animals were submitted to the following sequence of behavioral tests in a five-day setting: open field test, accelerating rotarod performance test, wire hang test, and pole performance test. Each cohort was humanely sacrificed after completing the behavioral test battery.

### 4.4. Open Field Test

The open field test was performed to assess the locomotor and exploratory activities according to previous publications [48]. Briefly, individual animals were placed in the center of a 50 cm × 50 cm open top arena under bright lighting and recorded with a ceiling-mounted video camera during a 10 min exploration time. The arena was divided into three areas: center area (30 × 30 cm, 36% of the total area), peripheral area and wall area. Distance travelled (cm), mobility rate (%) and time spent in the center (s) of the field x were measured using Ethovision XT 15 animal tracking software (Noldus Information Technology BV, Wageningen, The Netherlands). Mice that had a tendency of jumping onto the wall or spent >15 s rearing at the wall area were excluded from the final analysis.

### 4.5. Accelerating Rotarod Performance Test

The accelerating rotarod performance test was performed to assess the motor coordination over three consecutive days by using a rotarod apparatus (RotaRod 47650, Ugo Basile S.R.L., Gemonio, Italy). Each daily session started with a single 300-s training at a constant speed of 4 rpm. During the daily training session, mice that fell were returned to the rod immediately and the latency to the first fall and mean time of falls from the training session were recorded. After a 1h break, three consecutive 300-s accelerating rotarod (5–40 rpm) trials were performed with an interval of at least 30 min between each trial. For each trial, the latency to fall was recorded. A mouse clinging to the rod and completing a full rotation was regarded as a fall and the latency was recorded.

### 4.6. Pole Test

Mice were tested for their ability to descend a vertical pole (1 cm in diameter, 60 cm high) over one training and four consecutive test trials, separated by 5 min intervals. In brief, mice were placed at the top of the pole, facing upwards. Then, the total time of orienting themselves facing downwards and descending to the base of the pole was recorded. Data from test trials were averaged and used for analysis.

### 4.7. Wire Hang Test

The wire hang test was performed on the same day as the pole test to assess grip strength over three consecutive trials, separated by 5 min intervals. In brief, mice grasped onto a horizontally positioned wire (2 mm in diameter and 55 cm long), 35 cm above their home cage with bedding material to provide a gentle fall. The latency to fall was recorded and averaged for analysis for all three trials.

### 4.8. Tissue Collection and Processing

WT and zQ175^Δneo^ mice were sacrificed by cervical dislocation and transcardially perfused with ice-cold 0.1 M phosphate-buffered saline (PBS). Brains were removed, trimmed without olfactory bulbs and cerebellums and divided into two hemispheres. One hemisphere was kept in 4% paraformaldehyde (PFA) in 0.1 M PBS for immunohistochemical processing. The other hemisphere was micro-dissected to separate caudate-putamen, hippocampus and cerebral cortex, which were snap-frozen in liquid nitrogen and stored in −80 °C until further protein extraction. Testes were collected in all male animals. One testis was kept in 4% paraformaldehyde (PFA) in 0.1 M PBS, the other one was snap-frozen in liquid nitrogen until further processing.

### 4.9. Immunohistochemistry and Morphological Quantification

Formalin-fixed hemispheres were embedded in paraffin and cut into 4-μm-thick coronal sections using a rotation microtome (HM355S, Leica Biosystems GmbH, Nußloch, Germany) as described previously [75,76,77,204,205,206,207,208,209,210]. Sections (bregma +0.8 mm and −1.8 mm) were stained for microglia (IBA1, 1:1000, FUJIFILM Wako Chemicals Europe GmbH, 019–19741), astrocytes (GFAP, 1:500, Agilent, Santa Clara, CA, USA, Z033401-2) using a BOND-III^®^ automated immunostaining system (Leica Biosystems GmbH, Nußloch, Germany) with a hematoxylin counterstain (provided with the staining kit, Bond Polymer Refine Detection, DS9800). Sections for IBA1 staining were pre-treated with citric acid for 20 min before staining and for GFAP staining, the Bond Enzyme Pre-Treatment Kit (AR9551, Leica Biosystems GmbH, Nußloch, Germany) was applied to the sections for 10 min. After staining, tissue sections were digitized at 230 nm resolution per pixel using a Pannoramic MIDI II slide scanner (3DHISTECH Ltd., Budapest, Hungary). Quantitative analysis was performed automatically using deep-learning algorithms generated with the DeePathology™ STUDIO (DeePathology Ltd., Ra’anana, Israel) [76,77]. We generated algorithms to identify IBA1^+^ cells and GFAP^+^ cells. The algorithms were applied separately on the regions of interest (i.e., the caudate-putamen and cerebral cortex).

### 4.10. Histomorphologic Evaluation

To evaluate the CP and hemispheric atrophy, slides were stained with hematoxylin and eosin (H&E). A Pannoramic MIDI II slide scanner was used to obtain digital images of brain sections at 230 nm/pixel resolution. The CP (bregma +0.8 mm) and hemisphere (bregma −1.8 mm) sections were manually traced according to the Franklin & Paxinos mouse brain atlas [211] using the CaseViewer software (3DHISTECH Ltd., Budapest, Hungary), which also automatically calculated the area of the defined regions (cm^2^) of caudate-putamen and hemispheres. The areas were averaged with two sections of H&E staining per slide.

### 4.11. Western Blot Analysis

Mouse brain and testis tissues were homogenated with a bead mill homogenizer (SpeedMill PLUS, Analytik Jena GmbH, Jena, Germany). Tissue homogenates were lysed in ice-cold RIPA buffer (50 mM Tris-HCl, pH 8; 150 mM sodium chloride; 1 mM EDTA; 1% Triton X-100; 0.5% sodium deoxycholate; 0.1% sodium dodecyl sulfate), and complete protease inhibitor cocktail (Roche, Rotkreuz, Switzerland) for 30 min with constant vortex. Tissue lysates were centrifuged at 14,000 rpm for 30 min and the supernatants were collected. The final protein concentration in the supernatants was determined using the Pierce BCA assay kit (ThermoFisher, Waltham, MA, USA) and a total of 25µg protein was subjected to 7.5% SDS polyacrylamide gel electrophoresis (SDS-PAGE, ThermoFisher, Waltham, MA, USA), transferred onto a polyvinylidene difluoride (PVDF) membrane in transfer buffer (ThermoFisher, Waltham, MA, USA) after soaking in 15% methanol for 2 min. All membranes were blocked with 5% non-fat milk in phosphate-buffered saline containing 0.1% Tween-20 (PBS-T) and incubated with the anti-polyQ (1:1000; clone MW1, MerckMillipore, MABN2427) and anti-beta-tubulin (1:2500; clone AA2, MerckMillipore, T5076) on a shaker overnight at 4 °C, followed by appropriate horseradish peroxidase (HRP) secondary antibody (1:5000, Bethyl Laboratories, A90-216P) for 1 h in RT. Images were obtained by using the Octoplus QPLEX imaging system after incubating membranes with enhancer reagent (Clarity, ThermoFisher, Waltham, MA, USA). WB were repeated in at least three individuals from each genotype.

### 4.12. Statistical Analysis

All statistical analyses were performed using GraphPad Prism 9 software (GraphPad Software, San Diego, CA, USA). We verified the data for Gaussian normal distribution by using the Shapiro–Wilk normality test and for even variances using the Levene test. Detailed statistical methods are summarized in Table S3. Two-way analysis of variance (ANOVA) was performed to determine the significant differences between two genotypes (WT and zQ175^Δneo^), followed by Bonferroni’s post hoc test. For continuous repeated measurements, Student’s *t*-test was used to compare WT versus zQ175^Δneo^ mice. Data are presented as means ± standard deviation (SD). Differences were considered statistically significant when *p* < 0.05. N is reported in the figure legends and summarized in Table S3.

### 4.13. Compilation of the HD_MDS

The National Center for Biotechnological Information (NCBI; http://www.ncbi.nlm.nih.gov, accessed 1 October 2022) was searched for qualified compounds applying the key words ‘small-molecule’ and ‘Huntingtons’. References of the qualified reports were also searched for qualified references. Qualified molecular structures of compounds were retrieved either from PubChem (http://pubchem.ncbi.nlm.nih.gov, accessed 1 October 2022) or manually drawn using ChemDraw Pro version 20.1.1.125 (PerkinElmer Ltd., Beaconsfield, UK). Isomeric SMILES were considered where applicable and the SMILES codes were kept in the upper-case description scheme. CLogP, MW, MR, and TPSA were calculated applying SwissADME (http://www.swissadme.ch, accessed 1 October 2022) [85]. Of note, the given CLogP and TPSA values were calculated using an atomistic method [212] and a fragment-based method [213], respectively.

## Data Availability

Experimental and data files (HD_MDS) can be downloaded from http://www.doi.org/10.17605/OSF.IO/EJVWY (accessed on 21 November 2022). HD_MDS is also freely available on the www.panabc.info web page.

## Supplementary Materials

The following supporting information can be downloaded at https://www.mdpi.com/article/10.3390/ijms232314763/s1 or http://www.doi.org/10.17605/OSF.IO/EJVWY (accessed on 21 November 2022).

## Abbreviations

A_2A_R, adenosine receptor 2A; Aβ, amyloid-beta; ABC, ATP-binding cassette; ABC_BPMDS, ABC transporters-focusing binary pattern multitarget dataset; AD, Alzheimer’s disease; AMPK, AMP-activated protein kinase; BMI, body mass index; BTR1, bicarbonate transporter related protein 1; CASP, cysteine aspartase; ChE, choline esterase; ChR, choline choline receptor; CLogP, calculated octanol/water coefficient; COX2, cyclooxygenase 2; CP, caudate-putamen; CTX, cerebral cortex; C@PA, computer-aided pattern analysis; DAT, dopamine transporter; DMT-1, divalent metal transporter 1; DNA, deoxyribonucleic acid; DR, dopamine receptor; EAAT, excitatory amino acid transporter; ENT1, equilibrative nucleoside transporter 1; ESET, ERG-associated protein with SET domain FSH, follicle-stimulating hormone; GABAR, γ-amino butyric acid receptor; GFAP, glial fibrillary acidic protein; GLT-1, glutamate transporter 1; GLUT, glucose transporter; GSH, reduced glutathione; GnRH, gonadotropin-releasing hormone; HD, Huntington’s disease; HD_MDS, HD-focusing multitarget dataset; HDAC, histone deacetylase; 5HTRs, serotonin receptors; H&E, haematoxylin-eosin-stain; HRP, horseradish peroxidase; HSP, heat shock protein; 5-HTT, 5-hydroxy tryptamine transporter; HTT, human/murine huntingtin protein; *HTT*, human huntingtin gene; *Htt*, *Hdh* murine huntingtin gene; IBA1, ionized calcium-binding adaptor molecule 1; IL, interleukin; IUPAC, International Union of Pure and Applied Chemistry; KCC2, potassium-chloride co-transporter 2; KEAP, Kelch-like ECH-associated protein, KI, knock-in; LH, luteinizing hormone; LXR-α, liver-x-receptor α; MCT9, monocarboxylate transporter 9; MR; molar refractivity; MRI, magnetic resonance imaging; MSN, medium spiny neuron; muHTT, mutant huntingtin protein; MW, molecular weight; neo, neomycin; NCBI, National Center for Biotechnological Information; NET, norepinephrine transporter; NHE1, sodium-hydrogen exchanger 1; NKCC1, sodium-potassium-chloride co-transporter 1; NMDARs, *N*-methyl-_D_-aspartic acid receptors; NRF2, nuclear factor erythroid 2-related factor 2; OCTN2, organic cation/carnitine transporter 2; PBS, phosphate-buffered saline; PCR, polymerase chain reaction; PDE, phosphodiesterase; PDI, protein disulfide isomerase PFA, paraformaldehyde; pH, negative decadic logarithm of proton concentration; PHC, phosphate carrier protein; polyQ, polyglutamine; PPARGC1A, peroxisome proliferator-activated receptor γ coactivator 1α; PROTAC, proteolysis-targeting chimera; PKA, protein kinase A, ROCK, Rho-associated protein kinase, RNA, ribonucleic acid; ROS, reactive oxygen species; SD, standard deviation; SERT, serotonin transporter; SLC, solute carrier; SMILES, simplified molecular-input line-entry system; SDS-PAGE, sodium dodecyl sulfate polyacrylamide gel electrophoresis; SREBP2, sterol regulation element-binding protein 2; SVCT2, sodium-dependent vitamin c transporter; ThTr2, thiamin transporter 2; TPSA, topological polar surface area; UPRmt, mitochondrial unfolded protein response; UT1, urea transporter 1; VAChT, vesicular acetylcholine transporter; VGluT, vesicular glutamate transporter; VGSC, voltage-gated sodium ion channel; VIAAT, vesicular inhibitory amino acid transporter; VMAT2, vesicular monoamine transporter 2; WT, wild-type; ZnT10, zinc transporter 10; αRs, adrenoreceptors; σRs, sigma receptors.
