# Supplementary material for: A Novel Huntington’s Disease Assessment Platform to Support Future Drug Discovery and Development
Source: Int J Mol Sci. 2022 Nov 25;23(23):14763. doi: 10.3390/ijms232314763 (PMC9740291; doi:10.3390/ijms232314763)
Supplement: Supplementary file 1 [file ijms-23-14763-s001.zip › Supplementary Materials - Figure S1 - Table S2ab.pdf]

Supplementary Materials

https://doi.org/10.17605/OSF.IO/EJVWY

A Novel Huntington's Disease Assessment Platform to Support Future Drug Discovery and Development

Jingyun Wu [1], Luisa Möhle [1], Thomas Brüning [1], Iván Eiriz [1], Muhammad Rafehi [2], Katja Stefan [1], Sven Marcel Stefan [1,3,*], Jens Pahnke [1,3,4,5,*]

[1] Department of Pathology, Section of Neuropathology, Translational Neurodegeneration Research and Neuropathology Lab (www.pahnkelab.eu), University of Oslo and Oslo University Hospital, Sognsvannsveien 20, 0372 Oslo, Norway

[2] Institute of Clinical Pharmacology, University Medical Center Göttingen, Robert-Koch-Str. 40, 37075 Göttingen, Germany

[3] LIED, Pahnke Lab, University of Lübeck and University Medical Center Schleswig-Holstein, Ratzeburger Allee 160, 23538 Lübeck, Germany

[4] Department of Pharmacology, Faculty of Medicine, University of Latvia, Jelgavas iela 4, 1004 Rīga, Latvia

[5] Department of Neurobiology, The Georg S. Wise Faculty of Life Sciences, Tel Aviv University, 6997801, Tel Aviv, Israel

Figure S1. Rotarod performance tests at different ages. The figures show the rotarod performance at three consecutive days for animal at 15w - 57w of age (**A-G**). Rotarod performance test differences are first detectable between WT and zQ175$^{\Delta neo}$ at 36 weeks of age. Data are presented as mean ± SD; N = 5–6. Significance was calculated using two-way ANOVA with Bonferroni's multiple comparisons test and is given as *: p ≤ 0.05, **: p ≤ 0.01, ***: p ≤ 0.001, and ****: p ≤ 0.0001.

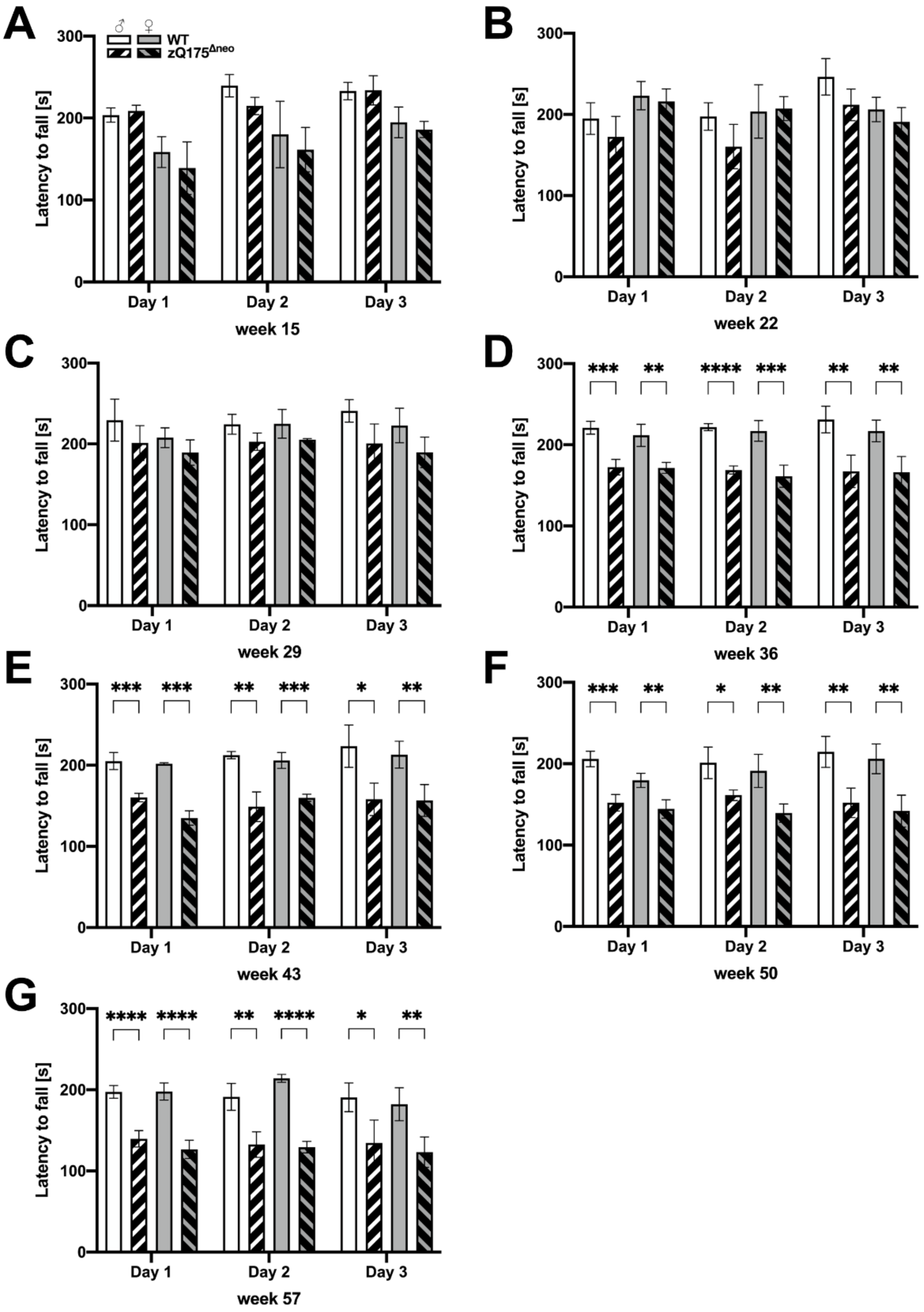

Table S1. Huntington's Disease Multitarget Dataset (HD_MDS)
Excel file can also be downloaded from https://doi.org/10.17605/OSF.IO/EJVWY or www.panabc.info .

Table S2. Sequences of primers for genotyping and repeat length determination.

a) PCR primers for genotyping.

PCR System	Prime name	Primer sequence
Mouse genotyping zQ175$^{\Delta neo}$	Fw	GGCTTATACCCCTACAGTAACAGTG
	Rv	TTCCAGGACAGCCAGAGCTA

b) PCR primers for zQ175 sequencing. PCR3 is the product which includes the CAG repeat region.

PCR System	Prime name	Primer sequence
PCR01	Hut_F01	TGCTAAGAGACAGGGCTCAC
	Hut_R01	AAACGCCAGGAGAGTACGAA
PCR02	Hut_F01	TGCTAAGAGACAGGGCTCAC
	Hut_intern_R02	TAAAGCCACCCTACACCTGG
**PCR03**	Hut_intern_F02	CAGGTCACTCTGGTTCCCTT
	Hut_R01	AAACGCCAGGAGAGTACGAA
	Seq-Primer	cgggcccaagatggctgagc

c) Sequencing information of the PCR03 product of the zQ175 line.
CAG repeats: 189 glutamines (polyQ)

```
>PCR03
caggtcactctggttcccttctcattgcagggagctcccagcacgctgcgttcgggaagctcaggccaccacctg
gcttgtggaaga[gagagctgctttgggtttcgggttccgagctccacaatcgctttcccggtgactccaggtgt
agggtggctttacgcaggaaaatttcttcgctgtcattcccctttccaacctttttcttccttccgggtctcccca
actcctctgcccacctcctcacttcttttctatcgctggtgccagggagccgccctaaagcccactctccgctca
gctccgtccctcatctagcagcccgccccgcccacctcatcctcttgcttggccctcttcactaagggggctgg
cttttgcgggaaggggcggggccacatcggcggggcggagagtcttaaactagcagaggccccgcaggcctgcgt
cctgacttcgggaaagaggacgacgcatccgcctgtcaattctgcgggtctggcgtggcctcgtctccgccggca
tgacgtcacgggacgcactcgccgcgagggttgccgggacgggcccaagatggctgagcgccttggttccgcttc
tgcctgccgcgcagagccccattcattgccttgctgctaagtggcgccgcgtagtgccagtaggctccaagtctt
cagggtctgtcccatcgggcaggaagccgtcatggcaaccctggaaaagctgatgaaggccttcgagtccctcaa
```

gtccttccagcagcagcagcagcagcagcagcagcagcagcagcagcagcagcagcagcagcagcaacagcagca
gcagcagcagcagcagcagcagcagcagcagcagcagcagcagcagcagcagcagcaacagcagcagcagcagca
gcagcagcagcagcagcagcagcagcagcagcagcagcagcaacagcagcagcagcagcagcagcagcagcagca
gcagcagcagcagcagcagcagcaacagcagcagcagcagcagcagcagcagcagcagcagcagcagcagcagca
gcagcagcagcagcagcaacagcagcagcagcagcagcagcagcagcagcagcagcagcagcagcagcagcagca
gcagcaacagcagcagcagcagcagcagcagcagcagcagcagcagcagcagcagcagcagcagcagcaacagca
gcagcagcagcagcagcagcagcagcagcagcagcagcagcagcagcagcagcagcaacagcagcagcagcagca
gcagcagcagcagcagcagcagcagcagcagcagcagcagcagcaacagcagccgccaccgccgccgccgccgccgcc
gcctcctcagcttcctcagccgccgccgcaggcacagccgctgctgcctcagccgcagccgcccccgccgccgcc
cccgccgccacccggcccggctgtggctgaggagccgctgcaccgaccgtgagtccgggcgccgcagctcccgcc
cgggccccgcgcccctggcctgcgtgctgggcatggccaacactgttccctgtccagagggtcgcggtacctccc
tgaggccaggctttcccggcccgggccctcgtcttgcggggtctctggcctccctcagaggagacagagccgggt
caggccagccagggactcgctgaggggcgtcacgactccagtgccttcgccgttcccagtttgcgaagttaggga
acgaacttgtttctctcttctggagaaactggggcggtggcgcacatgactgttgtgaagagaacttggagaggc
agagatctctagggttacctcctcatcaggcctaagagctgggagtgcaggacagcgtgagagatgtgcgggtag
tggatgacataatgcttttaggaggtctcggcgggagtgctgagggcgggggagtgtgaacgcatccaatgggat
attctttttccaagtgacacttgaagcagcctgtgactcgaggcacttcgtactctcctggcgttt

Table S3. Information about statistical analyses and number of samples

A - Statistical tests used

B - Number of samples

Excel file can be downloaded from https://doi.org/10.17605/OSF.IO/EJVWY
